# Schola Medicinæ Bristol (Review)

**Published:** 1933

**Authors:** 


### Review

**Schola Medicinæ Bristol: Its History, Lecturers and Alumni, 1833-1933.** Pp. 115. Illustrated. Bristol: John Wright & Sons, Ltd. 1933. Price 2s. 6d.—The author's name does not appear on the title-page, but is disclosed in a "foreword" by the Dean of the Faculty of Medicine. From this we learn that Dr. George Parker is responsible for this timely and interesting book on the Medical School of which Bristol is justly proud. In less than twenty pages the author has given us a clear and succinct account of the origin and development of our School, and throughout his work there is evidence of that detailed and accurate research which we have learnt to expect from him. The account of the actual foundation of the Bristol Medical School is introduced by a reference to the conditions of medical teaching and the qualifications of students which led to the desirability of the formation of the School by "the combination of lecturers from various groups" which then existed in Bristol. It seems surprising now to recall that in 1879 it was decided that the School should only be affiliated to University College, and full incorporation did not take place till 1893. The largest part of the book is taken up by lists of students, which are of great interest to all past and present members of the School; unfortunately, the errors in the original records are sufficiently numerous to destroy much of their value as an authority for reference. We hope these may be corrected in a future edition. There are some excellent illustrations of buildings associated with the progress of the School from its inception to the present day. For this record of outstanding interest and lasting value the Bristol Medical School is greatly indebted to Messrs. John Wright & Sons Ltd., who have generously produced it, as a centenary gift to the School, entirely at their own cost.

---

**Long Fox Memorial Lecture.**—The Long Fox Memorial Lecture will be delivered by Mr. Cecil A. Joll, M.D., Ch.M., F.R.C.S., on Tuesday, 7th November, 1933, in the Physiological Lecture Theatre of the University of Bristol at 5.30 p.m. The Vice-Chancellor will preside. Subject: "Recent Advances in the Ætiology, Diagnosis and Treatment of Cancer."

